# Understanding the flow behavior around marine biofilms

**DOI:** 10.1016/j.bioflm.2024.100204

**Published:** 2024-05-29

**Authors:** Maria J. Romeu, João M. Miranda, Ed. D. de Jong, João Morais, Vítor Vasconcelos, Jelmer Sjollema, Filipe J. Mergulhão

**Affiliations:** a LEPABE - Laboratory for Process Engineering, Environment, Biotechnology and Energy, Faculty of Engineering, University of Porto, Rua Dr. Roberto Frias, 4200-465, Porto, Portugal; b ALiCE - Associate Laboratory in Chemical Engineering, Faculty of Engineering, University of Porto, Rua Dr. Roberto Frias, 4200-465, Porto, Portugal; c CEFT—Transport Phenomena Research Center, Department of Chemical Engineering, Faculty of Engineering, University of Porto, Porto, Portugal; d Department of Biomedical Engineering, University of Groningen, University Medical Center Groningen, Antonius Deusinglaan 1, 97 13 AV, Groningen, the Netherlands; e CIIMAR – Interdisciplinary Centre of Marine and Environmental Research, University of Porto, Terminal de Cruzeiros do Porto de Leixões, Av. General Norton de Matos s/n, 4450-208, Matosinhos, Portugal; f Department of Biology, Faculty of Sciences, University of Porto, Rua do Campo Alegre, 4169-007, Porto, Portugal

**Keywords:** Marine biofouling, Biofilms, Hydrodynamic conditions, Shear forces, CFD modelling, Biofilm structural parameters, OCT

## Abstract

*In vitro* platforms capable of mimicking the hydrodynamic conditions prevailing in natural aquatic environments have been previously validated and used to predict the fouling behavior on different surfaces. Computational Fluid Dynamics (CFD) has been used to predict the shear forces occurring in these platforms. In general, these predictions are made for the initial stages of biofilm formation, where the amount of biofilm does not affect the flow behavior, enabling the estimation of the shear forces that initial adhering organisms have to withstand. In this work, we go a step further in understanding the flow behavior when a mature biofilm is present in such platforms to better understand the shear rate distribution affecting marine biofilms. Using 3D images obtained by Optical Coherence Tomography, a mesh was produced and used in CFD simulations. Biofilms of two different marine cyanobacteria were developed in agitated microtiter plates incubated at two different shaking frequencies for 7 weeks. The biofilm-flow interactions were characterized in terms of the velocity field and shear rate distribution. Results show that global hydrodynamics imposed by the different shaking frequencies affect biofilm architecture and also that this architecture affects local hydrodynamics, causing a large heterogeneity in the shear rate field. Biofilm cells located in the streamers of the biofilm are subjected to much higher shear values than those located on the bottom of the streamers and this dispersion in shear rate values increases at lower bulk fluid velocities. This heterogeneity in the shear force field may be a contributing factor for the heterogeneous behavior in metabolic activity, growth status, gene expression pattern, and antibiotic resistance often associated with nutrient availability within the biofilm.

## Highlights

• Biofilm development was higher at the lowest shaking frequency.
• Rougher biofilms induce larger perturbations in flow velocities around biofilms.
• Shear rate field was more heterogeneous for biofilms formed at the lowest rotation.
• Shear rate heterogeneity may contribute to heterogeneous cell behavior in biofilms.

## 1 Introduction

*In vitro* platforms have been used intensively to study biofilm development in different conditions [1]. While in some cases biofilm formation occurs in environments where the surrounding fluid is practically stagnant (for instance, in holding tanks in industry or particular biomedical applications), in other cases, significant fluid flow is observed. In these cases, it is essential to select adequate platforms capable of mimicking the hydrodynamic conditions prevailing in these environments so that reliable results can be obtained [2]. Our group has validated different platforms for these applications, including flow cells [3,4], flow chambers [5,6], microfluidic platforms [7], and agitated microtiter plates (MTPs) [[8], [9], [10]]. In order to characterize fluid behavior inside these platforms, we have used Computational Fluid Dynamics (CFD) to estimate the shear forces and the flow velocity. CFD is a numerical simulation technique that allows the assessment of different fluid flow parameters at a relatively low cost and faster than experimental procedures [11]. CFD offers a significantly enhanced level of flow field data compared to traditional experiments, as it generates results from a greater number of flow points [12]. Different CFD approaches have been employed to understand how flow patterns affect biofilm formation [13].

Biofilm formation in marine environments can have several negative impacts. There are economic problems related to the increased frictional drag on ship hulls and fuel consumption [14], and biofouling may impose environmental [15], ecological [[16], [17], [18]], and public health risks [19]. Additionally, microbiologically influenced corrosion may lead to the failure of different submerged structures or materials in contact with water [20]. Biofouling in marine environments starts with the adsorption of dissolved organic molecules, followed by the development of mature microbial biofilms and finally the settlement of macrofouler organisms [21]. Cyanobacteria are the primary components of marine biofilms [[22], [23], [24]] and release substantial quantities of extracellular polymeric substances [25,26], which play a vital role in the cohesion, stability, and structure of biofilms [27]. As a result, these microorganisms potentially govern the organization and functionality of biofilms [21,28]. Furthermore, it has been suggested that cyanobacteria can form symbiotic associations with diatoms [29,30], and the formation of biofilms by both bacteria and algae is essential. For marine environments, CFD approaches have been used to predict the effect of biofouling on the increased drag on ship hulls and the performance of marine vehicles [[31], [32], [33]].

In most CFD studies aiming to characterize the flow behavior inside biofilm reactors, simulations are made for the initial phases of biofilm development, where the hydrodynamics are unaffected by the biofilm's presence [34,35]. These initial events correspond to the surface conditioning phase, initial cell adhesion, and microcolony formation [36]. Results obtained from these studies are particularly useful as they predict, for instance, the shear forces that initial adhering cells must withstand. Previous work from our group has shown that agitated 12-well microtiter plates can mimic the hydrodynamic conditions found in marine environments [10]. The same reactor was later used to predict the antifouling behavior of marine coatings, which was also validated by field testing [37], showing the importance of biofilm development in marine biofouling. In this work, we go a step further, and we have evaluated the shear forces and flow velocity at a later stage in biofilm development, aiming to assess the shear rate distribution on a biofilm in marine environments. We observed not only the impact of hydrodynamics on biofilm development but also characterized the flow on the top of the biofilm layer using CFD. We show that shear forces vary in different areas of the biofilm, which may be a contributing factor to the physiological heterogeneity found within these bacterial communities.

## 2 Materials and methods

### 2.1 Organism and inocula preparation

Two cyanobacterial strains were used in this study and these were obtained from the Blue Biotechnology and Ecotoxicology Culture Collection (LEGE-CC) located at CIIMAR, Matosinhos, Portugal [38]. *Nodosilinea* sp. LEGE 06133 was isolated from a sample scraped off a submerged stone in the tidal pool at Moledo beach, Portugal (41.84963 N 8.866717 W). *Lusitaniella coriacea* LEGE 07167 was isolated from a sample scraped off a rock in the tide puddle at Lavadores beach, Portugal (41.12919 N 8.668578 W) [38]. Cells were grown in 750 mL culture in Z8 medium [39] supplemented with 25 g/L of synthetic sea salts (Tropic Marin) and B_12_ vitamin (Sigma Aldrich, Merck, Saint Louis, MO, USA). Cultures were incubated with a cycle of 14 h of light (10–30 μmol photons m^−2^ s^−1^) followed by 10 h of darkness at a temperature of 25 °C [10]. Cyanobacterial strains from *Lusitaniella* genus were found in marine environments in Portugal [40]. Conversely, strains from *the Nodosilinea* genus are present in dissimilar geographies, including different continents (Europe and Antarctica) and specimens can also be found in different ecologies, such as brackish water, freshwater, marine, and terrestrial environments [38]. Therefore, the widespread presence of these organisms underscores the significance of studying their biofilm formation behavior.

**Fig. 1:**
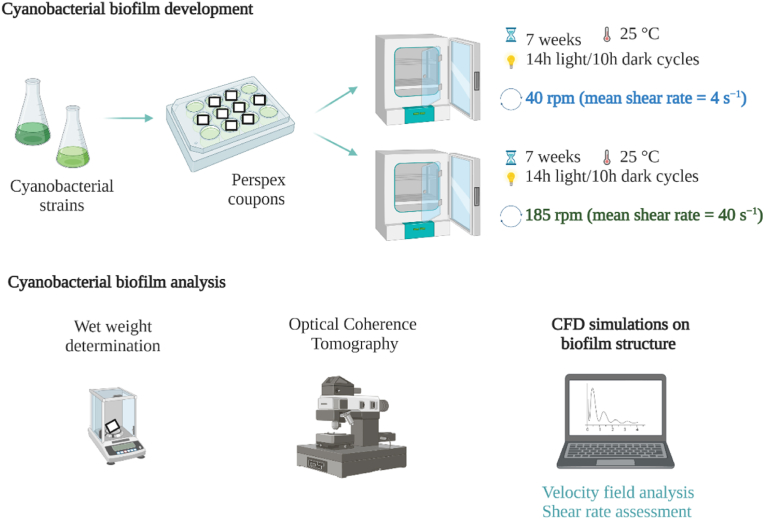
**Flowchart of the experimental stages of the current study.** The present study comprized a) the formation and development of cyanobacterial biofilms over 7 weeks (49 days) under two different rotation frequencies (40 and 185 rpm) (top), and b) weekly analysis of cyanobacterial biofilms by Optical Coherence Tomography (OCT) and wet weight determination (bottom). CFD simulations on biofilm structures were performed for 49-day biofilms, and the velocity and shear rate fields were determined.

The diagram in Fig. 1 illustrates the experimental procedures detailed in the following sections.

### 2.2 Biofilm formation

Perspex was used as a model surface for this study because it is commonly found in submerged marine artificial devices and equipment, such as hydrographic sensors and measuring devices (lenses, housings, underwater cameras), underwater windows of boats, aquaculture equipment, flotation spheres, and moored buoys [[41], [42], [43]]. Perspex (Neves & Neves, Lda, Portugal) coupons (1 cm^2^) were sterilized through immersion in a 2 % (v/v) TEGO 2000® solution (JohnsonDiversey, Northampton, UK) for 20 min under agitation (150 rpm). After they were rinsed with sterile distilled water to eliminate any potential residue from the disinfectant solution, coupons were subjected to sterilization for 30 min using ultraviolet (UV) radiation. Prior to biofilm development, the initial weight of each coupon was determined [10]. Biofilm formation was assessed on 12-well microtiter plates (VWR International, Carnaxide, Portugal) under optimized conditions for cyanobacterial growth. Transparent double-sided adhesive tape was used to fix the coupons, and once the tape was placed in the wells, all plates were subjected to UV sterilization for 30 min, after which the sterile coupons were fixed.

Each cyanobacterial suspension was adjusted to a chlorophyll *a* concentration of 1.5 μg/mL since the determination of this pigment concentration is commonly used as a biomass indicator in aquatic environments [10,44]. Briefly, cyanobacterial cells were harvested by centrifugation (3202×*g,* for 5 min at room temperature), and a volume of 2 mL of 99.8 % methanol (Methanol ACS Basic, Scharlau Basic, Barcelona, Spain) was added for chlorophyll extraction. After incubation for 24 h at 4 °C in the dark, samples were centrifuged again, and the supernatant was transferred to a glass cuvette. Absorbance measurements were performed at three wavelengths: 750 nm (turbidity), 665 nm (chlorophyll *a*), and 652 nm (chlorophyll *b*) using a V-1200 spectrophotometer (VWR International China Co., Ltd., Shanghai, China). Values were used to determine the chlorophyll *a* concentration (μg/mL) using Equation (1) [45]:

(1) $$Chl\;a\;\left(\mu g/\text{mL}\right)=16.29\times A^{665}-8.54\times A^{652}$$

These measurements were obtained in triplicate and dilutions were performed in Z8 medium with 25 g/L of synthetic sea salts and vitamin B_12_. A volume of 3 mL of adjusted cyanobacterial suspension in Z8 medium (prepared as described above) supplemented with nutritional factors, which support cyanobacterial growth [39], was inoculated per well.

Microtiter plates were incubated at 25 °C in an orbital shaker with a 25 mm orbital diameter (Agitorb 200ICP, Norconcessus, Portugal) at 40 rpm and 185 rpm. Previous work has shown that this platform mimics hydrodynamic conditions that prevail in real aquatic environments since the values achieved include the value of the shear rate estimated for a ship in a harbor, 50 s^−1^ [46]. Moreover, it was also shown to predict the biofouling behavior observed upon immersion in the sea for prolonged periods [37]. Briefly, to attain the shear rate values that can mimic aquatic environments, such as those found in a ship hull in a harbor and in partially submerged or even moored equipment and devices, microtiter plates were incubated under a rotation frequency of 185 rpm (achieving a mean shear rate of 40 s^−1^, [10]). To assess the flow behavior on biofilm development and structure, cyanobacterial biofilm formation at a lower rotation frequency (40 rpm, in which a mean shear rate of 4 s^−1^ is achieved) was also tested.

Biofilm development was followed for seven weeks (49 days) since it is accepted that a two-month interval for maintenance is the minimum duration for economically viable underwater monitoring systems [10,42]. The medium was replaced twice a week, and to mimic real light exposure periods, a photoperiod of 14 h light (8–10 μmol photons m^−2^ s^−1^)/10 h dark cycles) was applied.

### 2.3 Biofilm analysis

Sampling was performed every seven days and at each sampling day, two coupons containing biofilms developed by each cyanobacterial strain and under different rotation frequencies were analyzed. The culture medium was carefully removed, and it was replaced by 3 mL of sterile sodium chloride solution (8.5 g/L) to perform a washing step, eliminating loosely attached cells. The wells were filled again with 3 mL of sterile sodium chloride solution to determine biofilm thickness and biovolume by OCT. To complement the characterization of cyanobacterial biofilms, the determination of their wet weight was also performed over the 49 days, and images acquired by OCT on the last sampling day were used to perform the CFD analysis.

#### 2.3.1 Optical Coherence Tomography

3D images from cyanobacterial biofilms developed under the different rotation frequencies were captured and analyzed as reported by Romeu *et al.* [10], using a Thorlabs Ganymede Spectral Domain Optical Coherence Tomography system with a central wavelength of 930 nm (Thorlabs GmbH, Dachau, Germany). To avoid choosing a specific region of biofilm development, representative sections from the whole coupon surface were arbitrarily chosen. Assessment of biofilm thickness and biovolume was performed as described in detail on [60]. Since the axial resolution air/water obtained by this OCT device is about 5.8 μm/4.4 μm, only biofilm thicknesses over 10 μm were considered in this analysis. Briefly, the biofilm thickness and volume were obtained after Grey value thresholding using the well-established method of Otsu, which separates the background from the biofilm that connects with the surface. First, the bottom of the biofilm was determined as the best-fitting hyperboloid that connects the white pixels resulting from light reflection on the substratum surface. Subsequently, a grey-value threshold that separates the biofilm from the background was calculated based on the grey-value histogram of the entire image [48]. The automatic grey-value threshold suggested was assumed for most images. Moreover, even when a slight adjustment was required, the grey-value threshold was set up between 20 and 25. The upper contour line of the biofilm was defined as those pixels in the image that have a grey value just higher than the grey-value threshold and are connected to the bottom of the biofilm by pixels with grey values higher than the grey-value threshold. The image was then converted to a binary black and white image, and objects not connected to the bottom were rejected from the biofilm structure. The mean of biofilm thickness (${\bar{L}}_{F}$) was calculated as a function of the number of pixels (or voxels) between the bottom of the biofilm and the upper contour line for each vertical line in the image (${\bar{L}}_{F}$ is the mean of $L_{F,i}$ along the area described as $A_{x,z}$, where $L_{F,i}$ is the local biofilm thickness (μm) connected to the bottom at location *i* for each vertical *x,z* column (line of voxels). The biofilm roughness (R) is calculated as the standard deviation of the biofilm thickness (μm). Overall, a summary of the parameter definitions and equations is presented in Table 1.

**Table 1:** Variables and parameters definition for 3D OCT analysis.

Symbol/Parameter	Description	Formula
*x*	Pixel position on the horizontal axis (width)	*n.a.*
*y*	Pixel position on the vertical axis (height)	
*z*	Pixel position on perpendicular axis (depth)	
*i*	Index of *x*,*z* position in the horizontal plane	
$V_{vox}$	Volume of a voxel (μm^3^)	
$L_{F,i}$	Biofilm thickness at a given position *i* (μm)	
${\bar{L}}_{F}$	Mean biofilm thickness (μm)	
R	Roughness - standard deviation of biofilm thickness	
$A_{ROI}$	The total area of the ROI (mm^2^)	
${Acon}_{x,z}$	Number of connected voxels identified as belonging to biofilm matrix/bacteria in the biofilm (biovolume) in a horizontal plane at position *y*	
Biovolume	Number of all connected voxels in all images of a horizontal plane multiplied by the voxel size, providing an estimate of the biomass in the biofilm (μm^3^) per area of the ROI	$Biovolume\;\left(\frac{{\mu m}^{3}}{{\text{mm}}^{2}}\right)=\frac{\sum_{all\;planes}\left[\left({Acon}_{x,z}\right)\times V_{vox}\right]}{A_{ROI}\;}$

#### 2.3.2 Wet weight determination

The determination of cyanobacterial biofilm's wet weight was performed as reported by Romeu *et al.* [10]. After OCT analysis, the sterile sodium chloride solution was carefully removed from the wells, and the coupons were detached and weighed. The biofilm wet weight was obtained as the difference from the initial coupon weight determined prior to inoculation.

#### 2.3.3 CFD analysis

STL files obtained from OCT 3D images representing the biofilm structure, with a resolution of 4.9 μm/voxel, were processed to incorporate the domain boundaries. Each STL file contains a small frame out of the total captured image of 32.4 × 41.9 mm, that fully contains a biofilm and is constructed based on the calculated geometry (voxels) of the object that is connected to the bottom. This STL file was imported to OpenSCAD (openscad.org) and five additional boundaries were added: front, back, two lateral surfaces, and a top surface. When combined with the bottom boundary, which was formed by the original STL file of the biofilm, these elements constituted a 3D domain. This modified domain was then exported as a new STL file. The file was used to produce a 3D hexahedral mesh, refined in the proximity of the biofilm surface, by snappyHexMesh utility, a component of OpenFOAM, version 9 (OpenFOAM Foundation Ltd, UK) suitable for this purpose. Typically, the meshes have a maximum non-orthogonality of less than 60° and more than 90 % of the cells are hexahedral while the remaining ones are polyhedral. Complementary data about the velocity and the number of cells of each mesh used for all conditions can be assessed in Supplementary Material, Table S1).

The flow equations were solved in OpenFOAM using the simpleFoam solver. The transient flow equations were solved with a time step of 10^−6^ s. All cases reached a steady state for t < 0.08 s. For temporal discretization, the Crank-Nicolson method was used to ensure an implicit scheme for time evolution. For spatial discretization, the convective term was approximated using a Gauss linear scheme, while the diffusion term was handled by a Gauss linear corrected scheme. The Pressure-Implicit with Splitting of Operators (PISO) algorithm was selected to manage pressure-velocity coupling.

As for boundary conditions, a no-slip condition was imposed on the biofilm surface. At the top surface, located 1 mm above the bottom of the biofilm (y = 0), a velocity consistent with the average wall shear rate — previously calculated and reported in Romeu *et al.* [10] — was applied (Supplementary Material, Table S1). At the lateral boundaries, the slip condition for velocity was applied. Finally, at the front and back boundaries, both velocity and pressure gradients were set to zero. Shear rate values were calculated using the post-processing features of OpenFOAM and flow and shear rate fields were visualized using paraFoam, the OpenFOAM visualization utility.

### 2.4 Statistical analysis

All experiments were performed in duplicate (two biological assays with two technical replicates each). Data analysis was performed using the statistical program GraphPad Prism® for Windows, version 6.01 (GraphPad Software, Inc., San Diego, CA, USA) and results were compared using unpaired *t-*tests with a confidence level of 95 % (*; *P* < 0.05).

## 3 Results and discussion

**Fig. 2:**
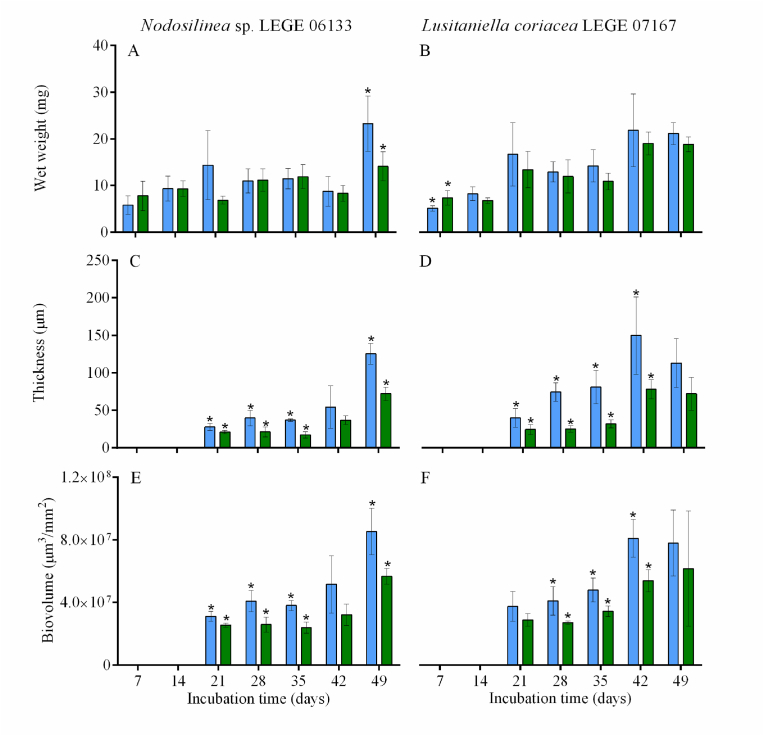
**Evaluation of cyanobacterial biofilm development on perspex for 49 days.** The parameters analyzed refer to wet weight (A, B), biofilm thickness (C, D) and biovolume (E, F). Biofilms were formed under rotation frequencies of 40 rpm (blue bars) or 185 rpm (green bars). Standard deviations from two biological assays with two replicates each, are represented. Symbol * indicates statistically different values between rotation frequencies for P < 0.05 (unpaired *t-*tests) at each incubation time. (For interpretation of the references to color in this figure legend, the reader is referred to the Web version of this article.)

Cyanobacterial biofilm development over 49 days is shown in Fig. 2. Values indicated in Fig. 2C–F are only presented from day 21, since for the first sampling days (days 7 and 14), biofilm thickness was below the OCT range. Overall, a cyanobacterial biofilm growth was observed over the seven weeks. Assessing the increase in biofilm growth from the initial (7 or 21 days) to the final sampling day (49 days), at different rotation frequencies and for both cyanobacteria, a higher increase was observed for biofilms formed at 40 rpm than those formed at 185 rpm. Indeed, for *Nodosilinea* sp. LEGE 06133, an increase of 75 % (wet weight), 78 % (thickness), and 64 % (biovolume) was determined for biofilms developed at 40 rpm, while increases of 45 %, 71 %, and 55 % were observed for the same parameters for biofilms developed at 185 rpm (Fig. 2A, C and E). Similarly, for *Lusitaniella coriacea* LEGE 07167, an increase of 76 % was observed on biofilm wet weight at 40 rpm, compared to 61 % obtained at 185 rpm (Fig. 2B). Regarding biofilm thickness and biovolume, values achieved under both rotation frequencies were very similar for this cyanobacterial strain (65 % *vs* 66 % for biofilm thickness and 52 % *vs* 53 % for biovolume, Fig. 2D and F).

Likewise, a higher biofilm development occurred at a lower rotation frequency (40 rpm) for both cyanobacterial strains (Fig. 2A–F). For *Nodosilinea* sp. LEGE 06133, biofilm mass, thickness, and biovolume obtained at higher rotation frequency (185 rpm) were, on average, 14 %, 40 %, and 33 % lower when compared to the values obtained at 40 rpm. For *Lusitaniella coriacea* LEGE 07167 these values (13 %, 50 % and 28 % for wet weight, thickness and biovolume, respectively) were similar to *Nodosilinea*. Overall, these results confirm that biofilm architecture (assessed here by the thickness and biovolume of the biofilm) is severely affected by the hydrodynamic conditions.

**Fig. 3:**
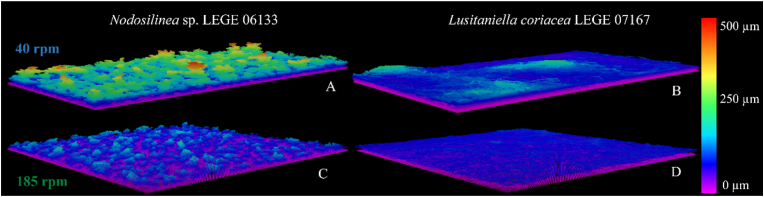
**3D OCT images of 49 days cyanobacterial biofilms formed on perspex at different rotation frequencies.** The color scale shows the biofilm thickness (μm). (For interpretation of the references to color in this figure legend, the reader is referred to the Web version of this article.)

Fig. 3 shows 3D OCT images of *Nodosilinea* sp. LEGE 06133 and *Lusitaniella coriacea* LEGE 07167 biofilms formed under different rotation frequencies after 49 days. Images show a higher biofilm development at the lowest shaking frequency for both strains. Also, it seems that the surface of the biofilms formed by *Nodosilinea* sp. LEGE 06133 is rougher. For this strain, and at 40 rpm, it was also possible to observe long streamers, reaching around 500 μm (Fig. 3A).

The presence of streamer structures in the biofilms of *Nodosilinea* sp. LEGE 06133 may be an adaptative strategy employed by this organism to allow nutrients and oxygen to penetrate the deeper layers of the biofilm by increasing the superficial area in contact with the surrounding environment. Additionally, it has been proposed that heterogeneity within a biofilm confers resistance against mechanical challenges [[49], [50], [51]].

**Fig. 4:**
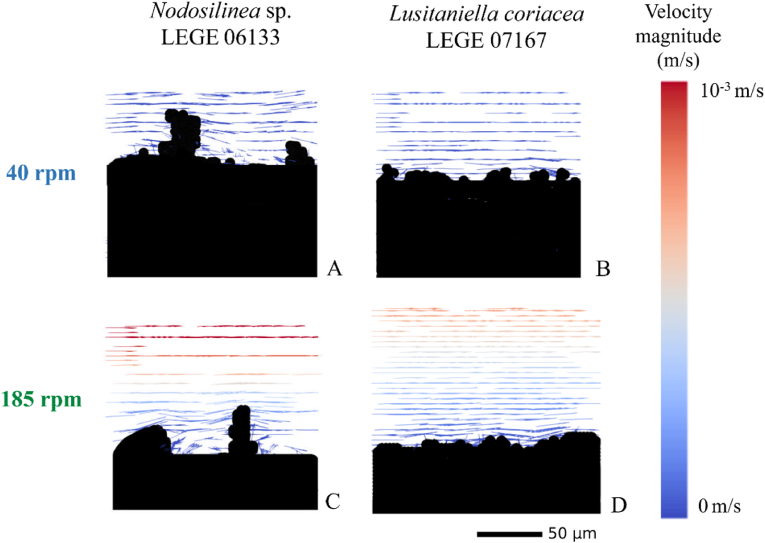
**Representative images of the velocity field in a cross-section of the top layer of cyanobacterial biofilms formed on perspex for 49 days at different rotation frequencies.** The black area represents the biofilm contour, whereas fluid velocity vectors are represented showing the direction of the flow. The color scale shows the velocity distribution magnitude (m/s). The biofilm scale is also represented (50 μm). (For interpretation of the references to color in this figure legend, the reader is referred to the Web version of this article.)

Using the 3D rendering of the biofilms obtained on day 49 by OCT, it was possible to design a mesh that could later be used in CFD simulations to analyze the flow behavior around the biofilms. Fig. 4 shows the velocity field on the top layer of cyanobacterial biofilms for both rotation frequencies. As expected, fluid velocities are higher at 185 rpm, indicating that mixing improves with the increase in rotation frequency (Fig. 4C and D). The highest fluid velocities are obtained around *Nodosilinea* sp. LEGE 06133 biofilms formed at 185 rpm (Fig. 4C). Since the surface of this biofilm has more irregularities than the one formed by *Lusitaniella coriacea* LEGE 07167 (Fig. 4D) these depressions reduce the surface effect on the flow in regions located further away from the biofilm. Also, for the former biofilm (Fig. 4C), it is possible to see the formation of eddies around the biofilm streamers in regions where the flow velocity is also very low. For *Lusitaniella coriacea* LEGE 07167 at 185 rpm (Fig. 4D), fluid velocities far away from the biofilm surface are lower, and the formation of eddies close to the biofilm surface is not observed. Eddies are also not visible close to the surface of biofilms from the same strain obtained at 40 rpm (Fig. 4B). This can be explained by a smoother biofilm surface compared to the biofilms from *Nodosilinea* sp. LEGE 06133. Closer to the biofilm surface, fluid velocities are lower at 40 rpm, as expected.

**Fig. 5:**
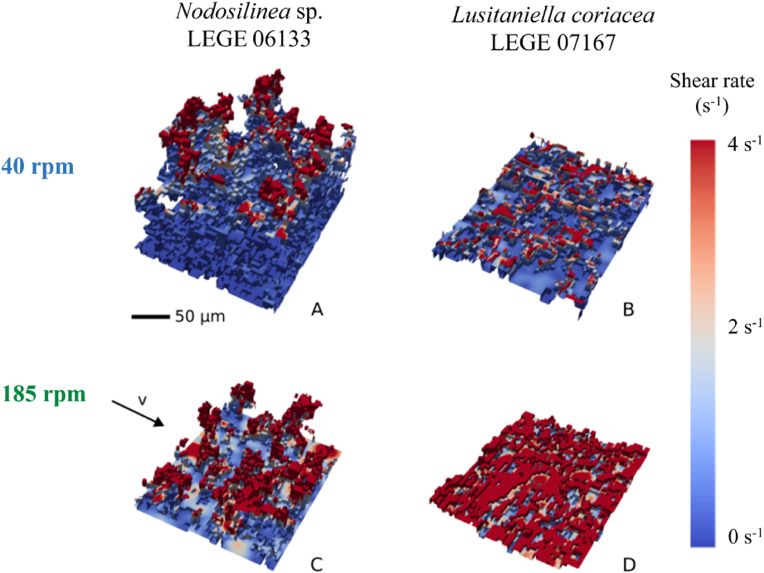
**Representative images of shear rate distribution on the top layer of 49 days cyanobacterial biofilms formed on perspex at different rotation frequencies.** The color scale shows the shear rate distribution (s^−1^). Values above 4 s^−1^ are represented in dark red. Complementary data about the distribution of the shear rates for each condition can be assessed in Supplementary Material, Fig. S1. The flow direction and the biofilm scale (50 μm) are also represented. (For interpretation of the references to color in this figure legend, the reader is referred to the Web version of this article.)

Since cyanobacterial biofilm growth was lower at 185 rpm (Fig. 2A–F, Fig. 3C and D), this may be a result of the higher velocity magnitude observed at this condition, which may promote biofilm sloughing and detachment over time, given the potential increase in shear forces. To clarify that, we have determined the shear rate distribution on the top layer of cyanobacterial biofilms formed after 49 days under both rotation frequencies (Fig. 5). It is possible to see that biofilm areas located at higher thickness values are exposed to higher shear forces and that shear rate variation seems to be higher for biofilms formed by *Lusitaniella coriacea* LEGE 07167 at the lowest shaking frequency (Fig. 5B). A more consistent pattern field of shear rate distribution and higher values were achieved for the flatter and homogeneous biofilm (Fig. 5D). Moreover, the higher shear rate values correspond to the layers of biofilm totally exposed to the surrounding environment (Fig. 5D) since streamer structures in heterogeneous biofilms (Fig. 5A and C) can protect the adjacent biofilm layers from these increased shear values. Through this analysis, it is also possible to observe the importance of stress distribution on the biofilm structure and identify regions prone to detachment. Previous studies have demonstrated that biofilm streamers, which can be spatially organized in different patterns, may act as precursors to the development of mature and denser biofilms [52]. Possibly, from data obtained in the present study, biofilm detachment begins from these higher streamers, which are subjected to higher shear values.

In previous works, we have conducted a proteomic analysis of biofilms formed at the same shaking frequencies. In one of these works [53], we used an unidentified filamentous cyanobacterium LEGE 06007 [38] isolated from a wave-exposed rock and found that the expression of a superoxide dismutase (A0A522XDZ7) was greatly enhanced at 40 rpm, whereas the expression of an uncharacterized protein with a disordered domain (A0A522XFY9) was increased at 185 rpm. In another study, we used a previously unidentified filamentous Synechococcales LEGE 06021 [38] that was later confirmed to be part of the *Toxifilum* genus. In that study [47], it was possible to identify several proteins, including one transketolase, one dehydratase, a ribosomal protein, and a transcription termination and a substrate-binding protein that were differentially expressed between the two hydrodynamic conditions used in this work. Although the strains used in the present work were not analyzed regarding their proteomic profile, it is likely that differential protein expression occurs within the biofilm, given the range of shear rate values that different cells are exposed to. Thus, we speculate that biofilms from *Lusitaniella coriacea* LEGE 07167 obtained at the lower shaking frequency may present a higher variability regarding their proteomic profile when compared to those formed by *Nodosilinea* sp. LEGE 06133. Also, it is possible that biofilm cells located at the streamers have a very different proteomic profile compared to the protein expression from their counterparts located at the base of the streamers, judging from the shear distribution obtained (Fig. 5). When analyzing small-scale shear effects on heterocystous cyanobacteria, Moisander *et al*. [59] have reported that shear (in the range of 2.2–18 s^−1^) had a negative effect on nitrogenase activity, CO_2_ fixation and affected the filament length of one of the *Nodularia* strains tested.

**Fig. 6:**
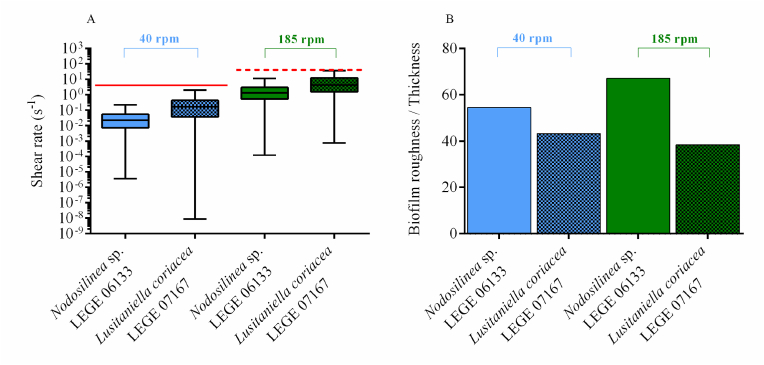
**(A) Box plot distribution of the shear rate values and (B) normalized roughness obtained for cyanobacterial biofilms formed after 49 days developed under different shaking frequencies.** Regular patterns in box plots (A) and bars (B) represent biofilms developed by *Nodosilinea* sp. LEGE 06133, whereas the dotted pattern represents biofilms from *Lusitaniella coriacea* LEGE 07167. A – Box plot showing the distribution of shear rate values from the minimum (lower whisker) to the maximum value not considered as an outlier (upper whisker). Complementary data about the frequency distribution of all shear rate values for each condition (demonstrating that excluded outliers represent residual values), can be seen in Supplementary Material, Fig. S2. The box is limited by the first (lower) quartile and the third (upper) quartile, and the median of all values is represented by a solid black line inside it. The red solid line and the red dotted line represent the shear rates calculated for the bottom of clean wells (without biofilm) considering the wells of agitated 12-well microtiter plates in an orbital shaker with 25 mm orbital diameter at shaking frequencies of 40 and 185 rpm (average shear rates of 4 and 40 s^−1^, respectively). (For interpretation of the references to color in this figure legend, the reader is referred to the Web version of this article.)

In previous works, we have shown for both filamentous [10,33,47,54] and coccoid cyanobacteria [55,56] that biofilm formation was affected by the shear forces. On one hand, the results presented in Fig. 2 suggest that hydrodynamics affect biofilm development. On the other hand, CFD calculations, as presented in Fig. 4, Fig. 5, show that architecture also affects the flow behavior around biofilms and the shear forces acting on them. Thus, it seems that global hydrodynamics affect biofilm development and architecture, which in turn affects local hydrodynamics. To ascertain this effect, we plotted the shear rate values obtained in the four conditions tested in this study (Fig. 6A). In a previous study [10], we have shown that, under the operational conditions used in this work, the average shear rate achieved at 185 rpm before biofilm formation was 40 s^−1^ (value indicated in Fig. 6A by red dotted line). For 40 rpm, an average shear rate value of 4 s^−1^ was obtained before biofilm formation (represented in Fig. 6A by a solid red line). Although average values ranging from 0.04 to 0.33 s^−1^ are obtained for 40 rpm, minimum values as low as 9 × 10^−9^ s^−1^ and maximum values up to 1.99 s^−1^ are obtained (Fig. 6A). For the higher agitation frequency, average values of 5 s^−1^ are obtained, whereas minimum values ranging from 1 × 10^−4^ and 8 × 10^−4^ s^−1^ are reached and maximum values between 11 and 36 s^−1^ are obtained (Fig. 6A). It is also possible to see from this figure that for *Lusitaniella coriacea* LEGE 07167, the shear rate now determined is similar to the shear rate obtained prior to biofilm development (the top value of the box plot, comprising 75 % of the values, is close to the red line lines). This means that the effect of biofilm formation on the flow behavior is less pronounced than for *Nodosilinea* sp. LEGE 06133 at both shaking frequencies (as the deviations from the predicted value prior to biofilm formation are larger). Although the biofilm spatial structure is sometimes characterized by the biofilm interface roughness (defined as the standard deviation of the biofilm height), when studying the biofilm fingering phenomena, Young et al. [57] have proposed a different parameter calculated as the ratio between the standard deviation of the active layer thickness and its average. Although the determination of the active layer is outside the scope of this work, we have plotted the biofilm roughness/thickness ratio in Fig. 6B to have an idea of the normalized roughness. We observe that biofilms formed by *Nodosilinea* sp. LEGE 06133 have higher values of this parameter for both shaking conditions. We therefore postulate that rougher biofilms have a greater effect on local hydrodynamics when compared to the clean surface (prior to biofilm formation). Regarding the shear rate variation that is seen in Fig. 6A, we observe that, in general, higher variation ranges are obtained for the lowest shaking frequency (with a slightly higher value for *Lusitaniella coriacea* LEGE 07167). The shear rate distribution is much more homogeneous at the higher shaking frequency. Since it has been previously shown that shear rate variations can cause differential patterns of protein expression in cyanobacteria [47,53], it is also possible that larger differences in protein expression profiles may be found between cells located higher in the streamers *versus* those located at the base of the streamer, particularly at the lowest shaking frequency (Fig. 5A). Additionally, it is possible that when biofilms start to develop in dynamic systems, the first microcolonies are subjected to high shear forces, and later on, these cells will be exposed to different shear values regarding their positioning in the mature biofilm. It is also likely that during biofilm development, these cells change their metabolism and protein expression to comply not only with nutrient availability but also with the changing shear forces.

## 4 Conclusions

The integration of OCT imaging into computational fluid modelling is challenging because of the increased computational time as well as the need for surface smoothing, noise removal, and advanced image thresholding to construct a consistent 3D mesh. OCT is a non-invasive technique capable of mesoscale analysis and is valuable for analyzing biofilm features that are not adequately addressed by other techniques like confocal microscopy. The integration of these two techniques enabled us to assess the effect of hydrodynamics in biofilm development and also the effect of the developed biofilm architecture on the fluid behavior around biofilms. Although for the simulations performed in the present work, the biofilm surface has been assumed to be a rigid, static wall with v = 0, biofilms are considered viscoelastic films, and their surface can slowly flow and change its shape [58]. However, this change in shape can be neglected for short timescales. Since the viscosity of the biofilm is sufficiently high for the velocity of its surface to be considered zero, the simulations performed in this study can be regarded as accurate for the time scale where the biofilm conserves its shape. We conclude that the biofilm architecture affects the shear forces acting on the developing biofilm and that biofilm regions that are more exposed to the surrounding fluid (e.g. streamers) are exposed to higher shear forces, whereas other areas located at the base of these streamers are protected against this shear. The fact that shear rate distribution varies so much, particularly at low fluid velocities, may have implications on the physiology of cells located in different regions of the biofilm (possibly affecting metabolic activity, growth status, gene expression, and antimicrobial resistance) besides the differences in nutrient availability, which have been far better studied in the literature.

## CRediT authorship contribution statement

**Maria J. Romeu:** Writing – original draft, Visualization, Methodology, Investigation, Formal analysis, Data curation, Conceptualization. **João M. Miranda:** Writing – review & editing, Visualization, Software, Methodology, Investigation, Formal analysis, Data curation, Conceptualization. **Ed. D. de Jong:** Writing – review & editing, Software, Methodology, Investigation, Formal analysis, Data curation. **João Morais:** Writing – review & editing, Methodology, Investigation. **Vítor Vasconcelos:** Writing – review & editing, Resources, Funding acquisition. **Jelmer Sjollema:** Writing – review & editing, Resources, Funding acquisition. **Filipe J. Mergulhão:** Writing – review & editing, Writing – original draft, Supervision, Resources, Funding acquisition, Conceptualization.

## Declaration of competing interest

The authors declare that they have no known competing financial interests or personal relationships that could have appeared to influence the work reported in this paper.

## Data Availability

Data will be made available on request.
