# Supplementary material for: Understanding the flow behavior around marine biofilms
Source: Biofilm. 2024 May 29;7:100204. doi: 10.1016/j.bioflm.2024.100204 (PMC11214183; doi:10.1016/j.bioflm.2024.100204)
Supplement: Multimedia component 1 [file mmc1.docx]

**Supplementary Material**

**Table S1. Velocity and the number of cells of each mesh used for all conditions of this study.**

| **Condition** | ***V* (m/s)** | **Mesh: number of cells** |
| --- | --- | --- |
| *Nodosilinea* sp. LEGE 06133  40 rpm | 0.004 | 717 373 |
| *Nodosilinea* sp. LEGE 06133  185 rpm | 0.04 | 494 955 |
| *Lusitaniella coriacea* LEGE 07167  40 rpm | 0.004 | 622 215 |
| *Lusitaniella coriacea* LEGE 07167  185 rpm | 0.04 | 129 123 |





**Figure S1. Histogram of the frequency distribution of shear rate values on each condition represented as relative frequency (percentages).** The center of the last bin was chosen automatically according to the maximum value of each condition. The bin width assumes different values for each case: (A) 0.01 s^-1^ for *Nodosilinea* sp. LEGE 06133 biofilms formed at 40 rpm, (B) 0.1 s^-1^ for *Lusitaniella coriacea* LEGE 07167 biofilms formed at 40 rpm, (C) 0.5 s^-1^ for *Nodosilinea* sp. LEGE 06133 biofilms formed at 185 rpm, and (D) 2 s^-1^ for *Lusitaniella coriacea* LEGE 07167 biofilms formed at 185 rpm.


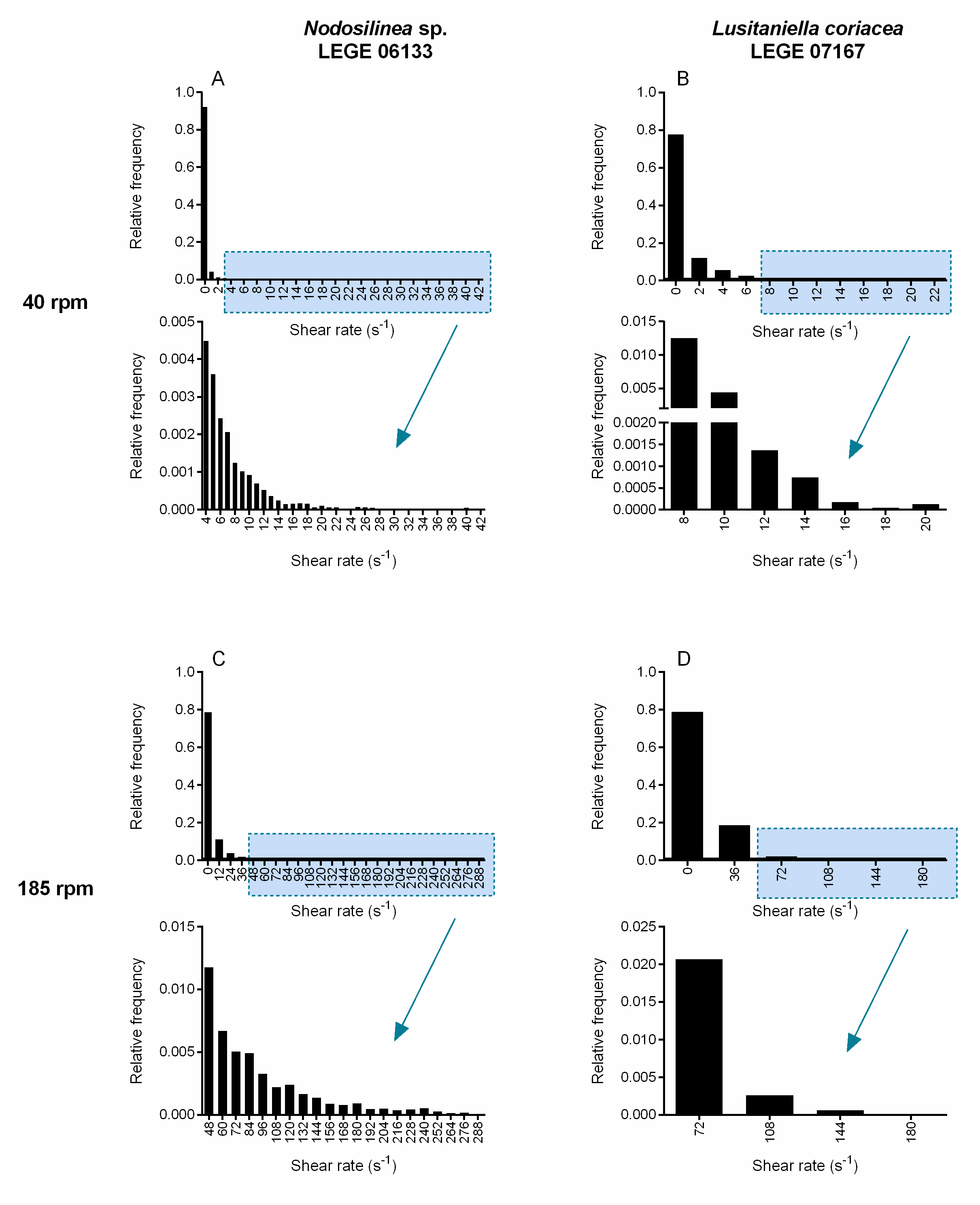
**Figure S2. Histogram of the frequency distribution of all shear rate values, on each condition represented as relative frequency (fractions).** The center of the last bin was chosen automatically according to the maximum values of each condition. The bin width assumes different values for each case: (A) 1 s^-1^ for *Nodosilinea* sp. LEGE 06133 biofilms formed at 40 rpm, (B) 2 s^-1^ for *Lusitaniella coriacea* LEGE 07167 biofilms formed at 40 rpm, (C) 12 s^-1^ for *Nodosilinea* sp. LEGE 06133 biofilms formed at 185 rpm, and (D) 36 s^-1^ for *Lusitaniella coriacea* LEGE 07167 biofilms formed at 185 rpm.
